# Biological benefits of collective swimming of sperm in a viscoelastic fluid

**DOI:** 10.3389/fcell.2022.961623

**Published:** 2022-09-22

**Authors:** Shiva Phuyal, Susan S. Suarez, Chih-Kuan Tung

**Affiliations:** ^1^ Department of Physics, North Carolina A&T State University, Greensboro, NC, United States; ^2^ Applied Science and Technology PhD Program, North Carolina A&T State University, Greensboro, NC, United States; ^3^ Department of Biomedical Sciences, Cornell University, Ithaca, NY, United States

**Keywords:** sperm motility, collective dynamics, rheotaxis, female reproductive tract, viscoelastic fluid

## Abstract

Collective swimming is evident in the sperm of several mammalian species. In bull (*Bos taurus*) sperm, high viscoelasticity of the surrounding fluid induces the sperm to form dynamic clusters. Sperm within the clusters swim closely together and align in the same direction, yet the clusters are dynamic because individual sperm swim into and out of them over time. As the fluid in part of the mammalian female reproductive tract contains mucus and, consequently, is highly viscoelastic, this mechanistic clustering likely happens *in vivo*. Nevertheless, it has been unclear whether clustering could provide any biological benefit. Here, using a microfluidic *in vitro* model with viscoelastic fluid, we found that the collective swimming of bull sperm in dynamic clusters provides specific biological benefits. In static viscoelastic fluid, clustering allowed sperm to swim in a more progressive manner. When the fluid was made to flow in the range of 2.43–4.05 1/sec shear rate, clustering enhanced the ability of sperm to swim upstream. We also found that the swimming characteristics of sperm in our viscoelastic fluid could not be fully explained by the hydrodynamic model that has been developed for sperm swimming in a low-viscosity, Newtonian fluid. Overall, we found that clustered sperm swam more oriented with each other in the absence of flow, were able to swim upstream under intermediate flows, and better withstood a strong flow than individual sperm. Our results indicate that the clustering of sperm can be beneficial to sperm migrating against an opposing flow of viscoelastic fluid within the female reproductive tract.

## Introduction

Collective swimming of sperm is widespread in mammalian species such as in cattle (Woolley et al., 2009; Nosrati et al., 2015; Tung et al., 2017), mice (Moore et al., 2002; Fisher and Hoekstra, 2010; Qu et al., 2021), opossums (Rodger and Bedford, 1982; Moore and Taggart, 1995), guinea pigs (Martan and Shepherd, 1973; Flaherty et al., 1993), and sheep (Creppy et al., 2013; David et al., 2015). Collective motion of sperm exists in various forms, including motile trains, massal motility, pairs, and dynamic clusters (Schoeller et al., 2020; Tung and Suarez, 2021). So far, the emergence of collective motion in mammalian sperm can be attributed to factors such as physical attachment (Moore and Taggart, 1995; Moore et al., 2002; Fisher et al., 2014), high concentrations of sperm (Schoeller et al., 2020), and viscoelasticity of the fluid in which sperm swim (Tung et al., 2017). For example, in the wood mouse (*Apodemus sylvaticus*), the sperm head has a hook that physically attaches to another sperm head or flagellum, resulting in motile sperm trains (Moore et al., 2002). These trains can be composed of hundreds to thousands of sperm, and they swim faster than individually swimming sperm. Several sperm with their heads conjoined together are also found to swim at a higher speed (Fisher and Hoekstra, 2010). In these cases of sperm physically attached to each other, the collective movement offers the advantage of moving sperm more quickly. Meanwhile, some collective dynamics of sperm do not require physical attachment between sperm. For example, massal motility is seen in undiluted samples of semen, where thousands to millions of sperm swim together to form mass wave-like motions in the fluid (Creppy et al., 2015). Sperm caught up in the wavelike motions have been associated with increased fertility outcomes (David et al., 2015), while the mechanism for such enhancement has remained unknown.

We previously reported dynamic clustering of bull sperm in a medium that mimics the viscoelasticity of some fluids in the female tract (Tung et al., 2017). Bull sperm in dynamic clusters are not physically attached to each other. When in a dynamic cluster, the sperm swim closely to each other and align in the same direction. Sperm freely leave and join various clusters over time (Tung et al., 2017). Unexpectedly, we found that the swimming speeds of sperm in clusters were not faster than the speeds of individually swimming sperm (Tung et al., 2017), leaving how this clustering behavior benefits sperm migration in the female tract unclear. Nevertheless, there may be other ways in which dynamic clustering may provide an advantage to migrating sperm in the female reproductive tract. In this study, we examined the progressivity of clustered sperm and the ability of sperm to swim against a flow of highly viscoelastic fluid, which occurs in the female reproductive tract (Suarez and Pacey, 2006; Lai et al., 2009; Suarez, 2016; Li et al., 2021).

Viscoelasticity occurs when a fluid contains components much larger than the solvent molecules, such as long polymers dissolved in water. A typical fluid that lacks viscoelasticity, such as a simple saline solution in water, does not retain a shape of its own, but rather acquires the shape of its container. Its viscosity is constant and independent of a stress applied upon the fluid. In contrast, the elasticity of a viscoelastic fluid, such as mucus that fills part of the female reproductive tract (Suarez and Pacey, 2006; Riley and Lauga, 2014; Li et al., 2021), varies depending on the stress applied on the fluid. Viscoelastic fluid also has a tendency to return to its previous shape within a short time scale when a stress is released (Barnes et al., 1989). *In vivo*, viscoelastic fluid flows in some parts of the female reproductive tract, such as mucus in the cervix, and sperm are required to swim against flows of viscoelastic fluid in order to fertilize the eggs (Suarez, 2016). Most previous studies of how fluid flow orients sperm migration or produces rheotaxis (swimming against a flow) (Bukatin et al., 2015; Zhang et al., 2016) have focused more on the flow of low-viscosity medium (Miki and Clapham, 2013; Tung et al., 2014, 2015a). Here, we aimed to examine how bull sperm, which have similar dimensions as human sperm, swim under flows of highly viscoelastic fluid, particularly whether sperm undergo rheotaxis to move against flows of viscoelastic fluid. We tested the hypothesis that *clustering of bull sperm increases the progressivity and rheotactic capabilities of sperm swimming in viscoelastic fluids.*

We used a previously developed microfluidic model (Tung et al., 2015a) that contains channels filled with a fluid that simulates the viscoelasticity of cervical mucus of cows in estrus (the fertile period of the bovine hormonal cycle). A syringe pump was used to provide well-controlled rates of fluid flow. It has been established that sperm swim in circular trajectories near a solid surface (Friedrich et al., 2010; Tung et al., 2015a), so we used the trajectory curvature of individual vs. clustered bull sperm under no flow as a quantitative tool to examine the progressivity of sperm movement. Further, we compared the responses of clustered vs individually swimming sperm in the presence of various rates of fluid flows.

## Materials and methods

### Media preparation

Tyrode Albumin Lactate Pyruvate (TALP) medium (Parrish et al., 1988) was prepared as a standard medium for bovine sperm. TALP medium is comprised of 99 mM NaCl, 10 mM HEPES free acid, 3.1 mM KCl, 0.39 mM NaH_2_PO_4_, 25 mM NaHCO_3_, 25.4 mM sodium lactate, 2 mM CaCl_2_, 1.1 mM MgCl_2_, 1 mM of sodium pyruvate, 5 mg/L of Gentamycin and 6 g/L of Bovine Serum Albumin (BSA). The final pH of the TALP medium was titrated to 7.42 with 1 N NaOH. Viscoelastic fluid was prepared by dissolving 0.7% of long-chain polyacrylamide (LC-PAM, 5–6 MDa) in TALP and with gentle magnetic stirring and alternating between room temperature and refrigeration for approximately 5 h, or until no clumps were observed within the fluid. This 0.7% PAM solution has comparable rheology to estrous bovine cervical mucus (Tung et al., 2015b). In preparation for experiments, the media were incubated at 38.5°C (bovine core body temperature) under 5% CO_2_ in humidified air for at least 2 h prior to adding sperm.

### Bovine sperm sample preparation

Frozen bovine semen provided by Genex Cooperative, Inc. (Ithaca, NY, United States) was extended in OptiXcell and transferred to 0.5 ml plastic straws (50 million sperm/straw) using their standard procedures (Kaproth et al., 2005). The straws were stored in liquid nitrogen. For sperm sample preparation, the straws were thawed in a 37°C water bath for 30 s. The thawed fluid was then centrifuged through two layers (40% and 80%) of Bovipure in Bovidilute solution, Spectrum Technologies, Inc., Healdsburg, CA, United States) at 300 x g for 10 min. Next, after removing the supernatant, the pellet was diluted in 3 ml of TALP and centrifuged at 300 x g for 3 min. The supernatant was removed and the sperm pellet was resuspended in 20 *μ*L TALP medium and incubated at 38.5 °C under 5% CO_2_ in humidified air. All the experiments were carried out independently using the two frozen semen straws from three different bulls.

### Microfluidic device fabrication

Silicon master mold fabrication was performed using the microfluidic design adapted from a previously developed microfluidic device (Tung et al., 2014). The silicon master consisted of a channel 4 cm long, 2.47 mm wide, and 120 *μ*m deep. The description of our microfluidic device setup can be found in Supplementary Figure S3. The casting of microfluidic devices in polydimethylsiloxane (PDMS) was as follows: 10:1 base to curing agent mixture of PDMS (SLYGARD 184 Silicone Elastomer kit, Dow Corning, Midland, MI, United States) were poured onto the fabricated silicon master, followed by degassing the PDMS mixture in an evacuated desiccator chamber for 30 min and curing at 65°C for 1 h. To make a sperm seeding port and a fluid input port, respectively, a 2 mm hole and a 1 mm hole were made in the PDMS pieces using Sklar Tru-Punch disposable biopsy punches (Sklar, West Chester, PA, United States). The PDMS pieces were bonded to glass slides using oxygen plasma cleaner (HARRICK PLASMA, PDC-32G, Ithaca, NY, United States) in a high RF power setting for 60 s. The microfluidic device channel was filled with viscoelastic 0.7% PAM in TALP medium and was equilibrated at 38.5 °C under 5% CO_2_ in humidified air before performing experiments.

### Addition of sperm to device

Two straws of frozen bovine semen (50 million sperm/straw) from one bull were used in each experiment. The sperm were prepared as described above. An aliquot of 5 µl sperm suspension was seeded 2–3 times to populate the device chamber. Sperm were allowed to swim out of the suspension into the viscoelastic medium for 30–45 min. Then videos were made of sperm swimming 3–5 mm from the seeding port, close to the center of the 2.47 mm wide channel. In this region of the channel, the sperm concentration was 2.95–5.54 million sperm/ml. Note that sperm were predominantly found on the channel surfaces, as expected, instead of being uniformly distributed.

### Flow ranges and experimental setup

**FIGURE 1:**
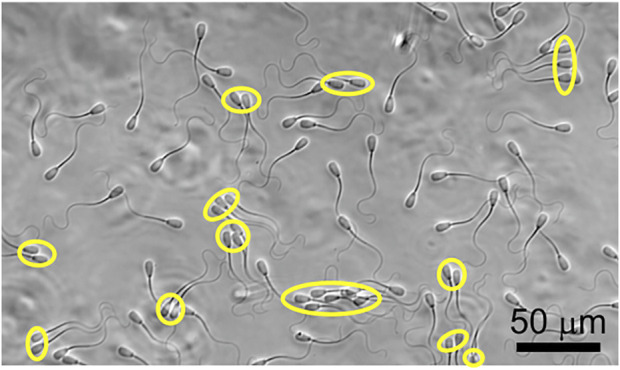
Coexistence of individually swimming and clustered sperm. Clustered sperm are labeled with yellow ovals.

Tubing (ETT-24, Weico Wire & Cable) connected to a 1 ml syringe (BD, Franklin Lakes, NJ, United States) was inserted into the 1 mm fluid inlet port of a microfluidic device filled with 0.7% PAM viscoelastic fluid and the device was placed in an on-stage environmentally controlled chamber (operated by OKO-Touch) heated to 38.5 °C and humidified to 65% on a Nikon Eclipse inverted microscope. Then, 5 *μ*l sperm suspension was seeded 2–3 times into the 2 mm seeding port, and the device was incubated for 30–45 min on the stage to allow sperm time to swim into the polymer solution until the sperm count was similar to that in Figure 1. This procedure was used to maintain the rheological properties of the polymer solution. Next, the syringe pump (KDS-230, KD Scientific, Holliston, MA, United States) was used to provide flow rates ranging from 0 to 5 *μ*l/min (equivalent to 0–13.5 1/s shear rates). The syringe pump was kept running for ≈60 s to establish a stable fluid flow condition during the experiment. Each experiment lasted 2–2.5 h.

### Analysis of sperm orientation and trajectory curvature

An Andor Zyla digital video camera and 20x objective (S Plan Fluor ELWD) were used to record phase-contrast images of sperm swimming on the lower surface of the microfluidic device chamber under a range of fluid flow rates. NIS Element BR software was used to control recording at 6.67 frames/sec and to view image files. The duration of each recorded video was 1 min. The recordings were used to compare movements of individually swimming sperm with those of sperm in clusters. To analyze sperm orientation at a given moment, all sperm in one still image were manually tracked using the straight-line tracking tools in ImageJ software. All sperm within a frame were tracked, and once sperm orientation reached a steady-state, several frames were analyzed to improve statistics.

To analyze individually swimming sperm trajectories, each sperm was manually tracked for 3.6 s using the Manual Tracking plugin in ImageJ and the tracks were plotted using MATLAB. For clustered sperm, one sperm out of each cluster of 2-4 sperm was manually tracked for 3.6 s, as described for individual sperm. This selection of small clusters was made for technical practicalities. First of all, we needed a trajectory to be long enough to see if it is curved or not. However, since sperm were not bound to each other in our dynamic clusters, they were free to dissociate from the cluster to become individual during the period being tracked. We found that sperm in clusters of more than 4 sperm often left the clusters in less than 3.6 s therefore could not be part of the analysis. The curvature of each trajectory was determined by fitting location data points (*x*, *y*) in to a circle (defined as *x* ^2^ + *y* ^2^ + *ax* + *by* + *c* = 0) to compute the radius of curvature by using the “fitnlm” function of MATLAB. The coordinate for the center of each circle was (*h*, *k*) = ( − *a*/2, − *b*/2) and the radius (*R*) was given by $R=\sqrt{\left(h^{2}+k^{2}-c\right)}$ . To get all points on the circumference of the circle, we used the parametric equation of a circle defined as: (*X*, *Y*) = (*R* cos *θ* + *h*, *R* sin *θ* + *k*), where *θ* = *atan*2(*y* − *k*, *x* − *h*), where atan2 is a MATLAB function for arc tangent (https://www.mathworks.com/matlabcentral/answers/559322-fitting-a-circle-with-fitnlm#comment_924827).

### Analysis of sperm clustering and responses to a flow

Two sperm were defined as swimming within the same cluster when their head orientations were within 20° and the sperm were separated by less than 17.5 *μ*m. This definition was consistent with previous work Tung et al. (2017). The overall behaviors were not sensitive to the exact definition. When we assessed videos of sperm swimming in a flow, some sperm swam upstream, some swam downstream, some were pushed back, and some were swept downstream. Each classification was made based on 80 consecutive frames (12 s) of the videos, and only live sperm cells were counted during the analysis. We categorized sperm as swimming downstream when they were swimming in the direction of the flow (Figure 5B, Supplementary Video S2) throughout the 12 s long video. Sperm were considered to be swept away when they initially swam against the fluid flow and their orientation changed toward downstream during the 12 s video (Figure 5C, Supplementary Video S3). Sperm were considered to be pushed back (Figure 5A, Supplementary Video S1) when they were not moving forward over the course of the 80 frames while maintaining upstream orientation. We chose this definition of pushed back to avoid repeated measurements of the same sperm. Since swimming downstream, swept downstream, or pushed back sperm were not moving forward against a flow, they would be jointly referred to as “failing in rheotaxis”. In each individual or clustered category, the percentages of rheotaxis failure were calculated by adding downstream swimming, swept downstream, and pushed back sperm, and dividing by the total individual or clustered sperm.

### Statistical analysis

All error bars denote standard errors of the mean (SEM) unless otherwise noted. *p*-values less than 0.05 were considered to be statistically significant. The differences in histogram distribution were calculated using the two-sample Kolmogorov Smirnov (K-S) test. A *t*-test was performed in Excel to detect differences between the means, and a two proportion z-test was conducted to find differences between two proportions. All data were analyzed using MATLAB software unless otherwise noted.

## Results

### In the absence of a flow, the swimming of clustered sperm was more directional than that of individually swimming sperm

**FIGURE 2:**
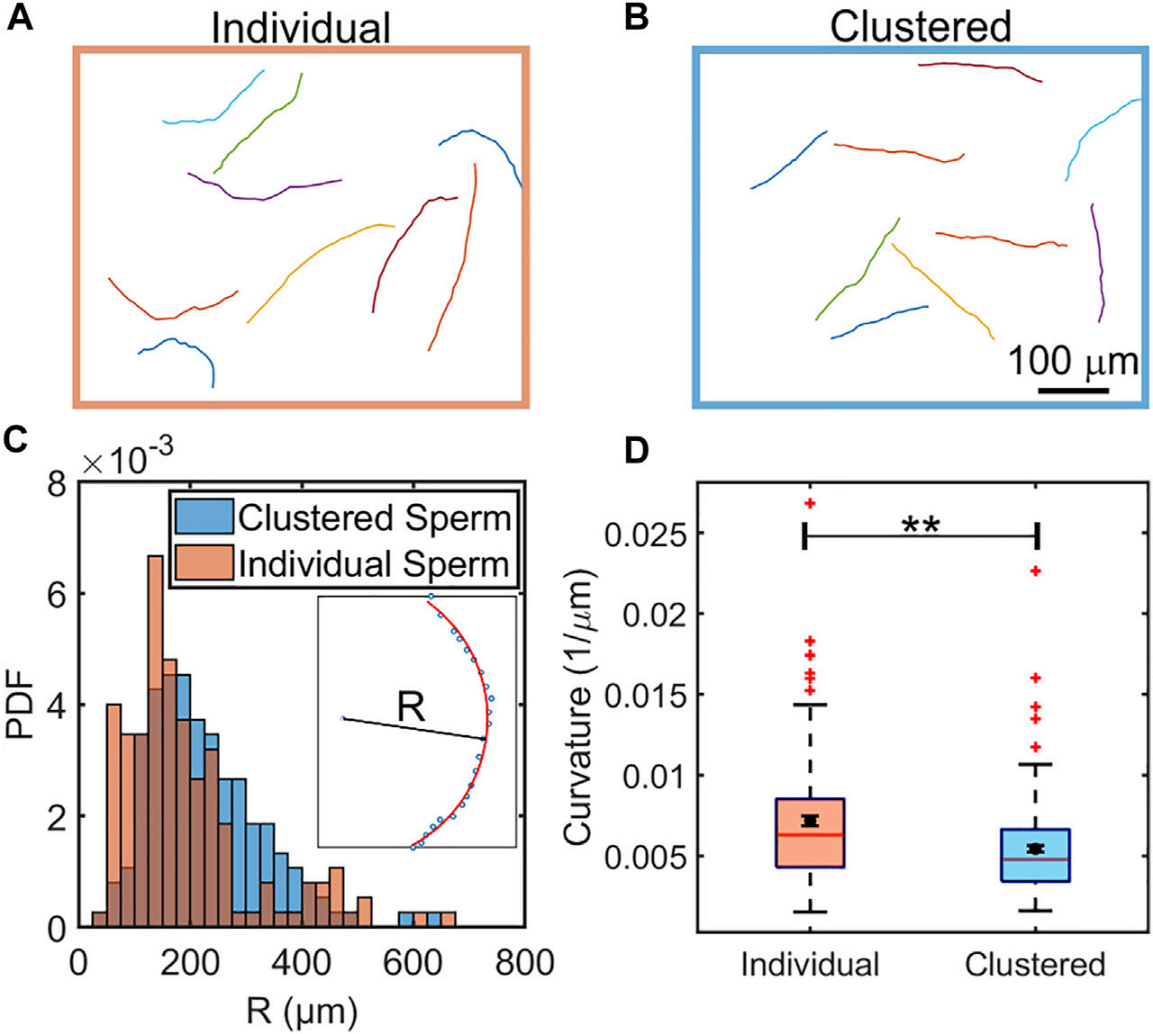
Comparison of swimming trajectories of individual **(A)** and clustered **(B)** sperm in the absence of flow. N = 9 sample trajectories, each sampled at 6.67 Hz and for 3.60 s. The distributions of radii of curvature of clustered (blue) and individual (orange) sperm from 150 trajectories each **(C)**. Inset: Calculation of the R was performed by fitting a trajectory to a partial circle using MATLAB. **(D)**, Box plot comparison of curvature of individual and clustered sperm. The means are shown by a black dot. The box plot shows the median, 25%, and 75% quartiles; whiskers show the smallest and largest data within 1.5 interquartile ranges below 25% and above 75% quartiles respectively; data beyond the whiskers are outliers and shown by +. N = 150 trajectories each sampled at 6.67 Hz and for 3.60 s. Error bars represent standard errors of the mean (SEM) and **indicates *p* < 0.01.

In Figure 1, we show the co-existence of individually swimming and clustered sperm, and compared the swimming trajectories of individually swimming and clustered sperm when there is no flow in Figures 2A,B. Note that, since roughly half of the sperm were found in clusters and the other half were found swimming individually at a given moment, the analyses on the two populations were obtained from the same videos. The majority of the clustered sperm comprised 2-4 cells, and each trajectory was 3.60 s long. The swimming track length of individually swimming sperm (Figure 2A) appeared to extend slightly further than the track length of clustered sperm (Figure 2B), which was verified by statistical analysis in Supplementary Figure S1 (*p* < 0.05 by two tailed *t*-test). This is in agreement with a previous study that clustered sperm on average swim at a slower speed than individual sperm (Tung et al., 2017).

Clustered sperm trajectories appeared to be more straight than those from individually swimming counterparts. To verify this, we computed radius of curvature (*R*) for each trajectory through curve fitting (Figure 2C inset), and then compared the distributions from the two populations (Figure 2C). We found that the two distributions of the radius of curvature (*R*) were significantly different (*p* < 0.001 by two sample K-S test). Likewise, the mean and median of the trajectory curvatures (1/*R*) of clustered sperm were also found to be less than those of individually swimming sperm (*p* < 0.01 by two tailed *t*-test), as shown in a box plot (Figure 2D). The comparison of radii of curvature (*R*) and the logarithm of radii (ln *R*) were likewise significantly different (*p* < 0.01 for *R* and *p* < 0.001 for ln *R* by two tailed *t*-test), as shown in Supplementary Figure S2. We concluded that, in a viscoelastic fluid, clustering enables sperm to swim straighter (more progressively) than when sperm swim individually.

### Emergence of sperm rheotaxis in a highly viscoelastic fluid differed from that of sperm in a low-viscosity fluid

**FIGURE 3:**
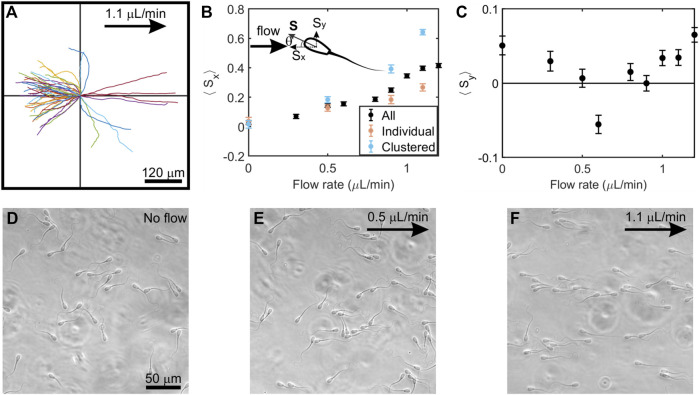
Behavior of sperm swimming in a low-speed flow of viscoelastic fluid. **(A)**, In a 1.1*μ*l/min flow, sperm showed linear trajectories when swimming both upstream (upper and lower left quadrants) and downstream (upper and lower right quadrants) (*N* = 50 trajectories; each trajectory is 4.5 s long) **(B)**, The vector mean of the orientation of all sperm, clustered, and individual sperm along the *x*-axis (⟨*S*_*x*_⟩) at increasing flow rates. Upper left: schematic of sperm head orientation denoted by a unit vector **S**, where *θ* is the angle to the *x*-axis. *S*_*x*_ and *S*_*y*_ is an orientation of sperm along the *x*-axis and *y*-axis respectively. ⟨*S*_*x*_⟩ = 0 implies random swimming along the *x*-axis while ⟨*S*_*x*_⟩ = 1 implies perfect alignment of sperm in *x*-axis. **(C)**, The vector means of the orientation of all sperm along the *y*-axis (⟨*S*_*y*_⟩) at increasing flow rates. Three experiments (n = 3) from semen samples of three bulls were carried out, and each point represents N ≈ 800–1,100 tracked sperm cells. Each data point in the figure denotes the mean of three experiments and error bars represent the standard errors of the mean. **(D)**, **(E)**, **(F)**, Photo micrographs (264 × 264 *μ*m) of sperm swimming at no flow, 0.5 *μ*L/min, and 1.1 *μ*L/min, respectively. Flows are applied toward the positive *x*-direction and denoted by an arrow.

Emergence of rheotaxis in sperm has been quantitatively explained by a hydrodynamic model (Kantsler et al., 2014; Tung et al., 2015a), so we examined whether a similar mechanistic model applies when sperm swim in a flow of highly viscoelastic fluid. Similar to sperm rheotaxis in a low-viscosity medium, sperm swam upstream within certain flow rates in a high-viscoelastic fluid, as shown in Figure 3A. However, some of the trajectories shown in Figure 3A were harder to reconcile with the existing mechanistic model (Tung et al., 2015a). According to the existing model, when sperm swim close to a solid interface in a flow, the broad sperm head experiences more hydrodynamic resistance than the narrow sperm tail. This causes the tail to swing around toward the downstream direction, thereby orienting the sperm to swim upstream. Under this model, sperm exhibit curved trajectories when swimming toward a downstream direction (while turning toward upstream), while upstream swimming sperm exhibit linear trajectories while swimming near a wall (Tung et al., 2015a). In the highly viscoelastic fluid, we observed linear upstream swimming trajectories. However, in contrast to the model, we also found sperm swimming downstream in linear trajectories in the highly viscoelastic fluid (Figure 3A). Whereas the number of these unexplained trajectories was not high (3 out of 50), their existence indicated that modifications are needed to the mechanistic model in order to account for linear downstream swimming. Note that, in our microfluidics device, nearly all sperm swim close to surfaces soon after they are introduced into the channel and we were tracking sperm that were swimming close to the bottom surface of the channel.

It has been understood that the onset of upstream swimming occurs in a low-viscosity fluid when a flow exceeds a certain threshold flow rate (Tung et al., 2015a). After the onset, the upstream trajectories become linear. We also show here that, unlike in low-viscosity fluid, there is no clear onset of the emergence of upstream swimming in highly viscoelastic fluid.

Figure 3B illustrates the average sperm orientation under various flow rates for clustered, individual, and all sperm. In the absence of flow, sperm appeared to swim in all directions in Figure 3D. We calculated the average sperm orientation by assigning a unit vector to the direction of each sperm, and then averaged the component of presumed upstream direction ⟨*S*_*x*_⟩ and the component in the perpendicular direction ⟨*S*_*y*_⟩. When there was no flow, ⟨*S*_*x*_⟩ = 0.03 ± 0.01 (mean ± SEM, N = 3,033), which agreed with the expectation of random orientation. ⟨*S*_*x*_⟩ gradually and steadily increased with a flow rate from 0.3 to 1.2 *μ*l/min.

From the sperm trajectories in Figures 2A,B, we found sperm orientation turned in both clockwise (CW) and counter-clockwise (CCW) directions (viewed from above), which was also a departure from what has been seen in low-viscosity medium (Miki and Clapham, 2013; Kantsler et al., 2014; Tung et al., 2015a). Applying the existing mechanistic model (Tung et al., 2015a), once sperm locked into the upstream (x) direction, we would anticipate seeing them swimming in either direction (±y) perpendicular to the flow. Indeed, upstream trajectories were found on both upper and lower quadrants to the left side of Figure 3A. Further, when we computed ⟨*S*_*y*_⟩, all values were close to 0 (Figure 3C).

### Clustered sperm exhibited better rheotactic responses than individual sperm

**FIGURE 4:**
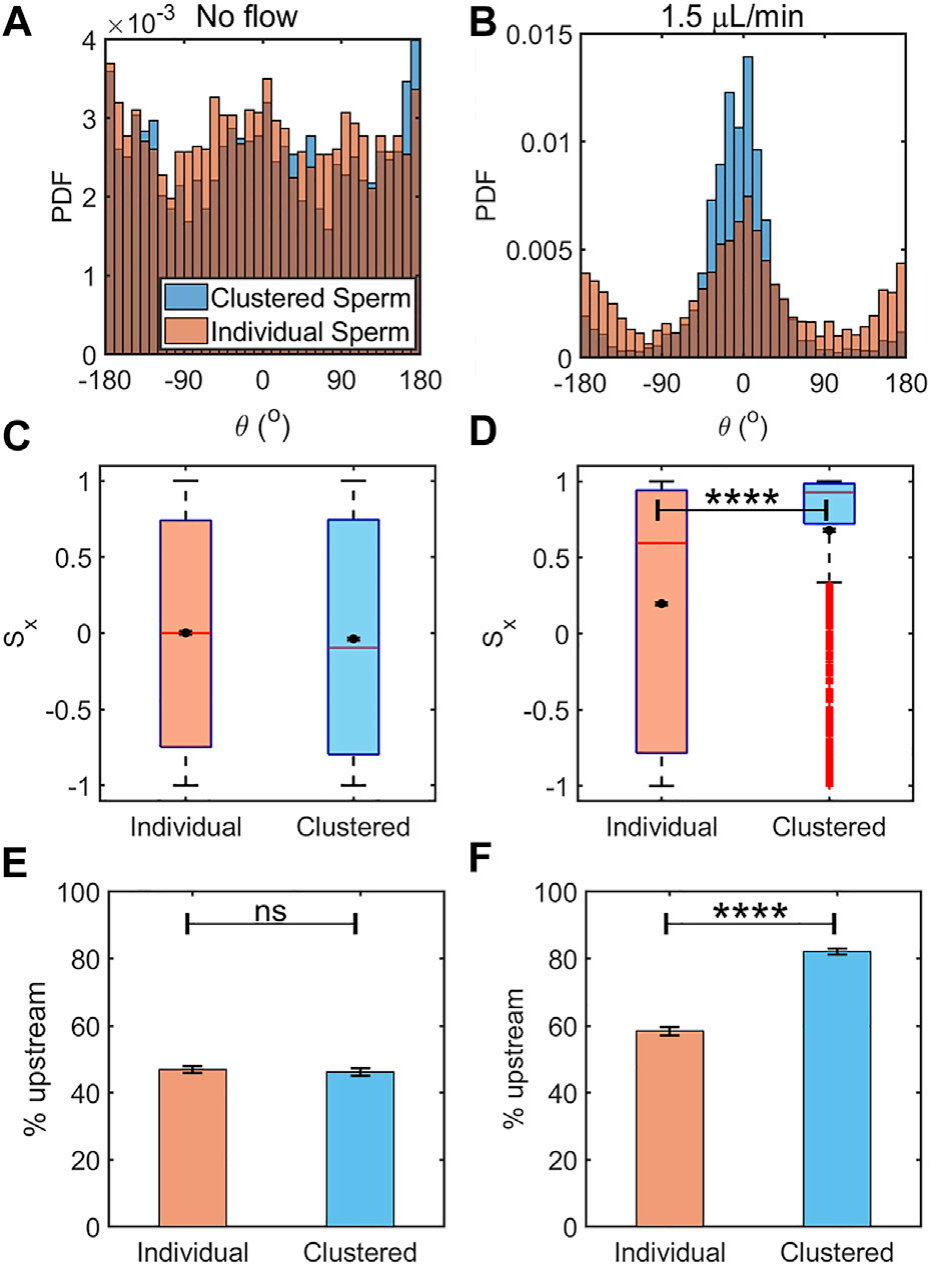
Clustered sperm exhibited greater rheotactic responses than individual sperm. Histogram of orientation angle between the clustered and individual sperm in the absence of flow **(A)** (N ≈ 2,800 cells for each) and under an intermediate flow rate of 1.5 *μ*L/min **(B)** (N ≈ 2,900 cells for each). Box plot along with mean of the sperm upstream orientation component (*S*_*x*_) under no flow **(C)** and an intermediate flow rate of 1.5 *μ*l/min **(D)**. The means are shown by a black dot. The box plot shows the median, 25%, and 75% quartiles; whiskers show smallest and largest data within 1.5 interquartile ranges below 25% and above 75% quartiles respectively; data beyond the whiskers are outliers and shown by +. Percentages of sperm oriented upstream, individual vs. clustered sperm under no flow **(E)** (N ≈ 4,300 cells) and flow rate of 1.5 *μ*L/min **(F)** (N ≈ 3,500 cells). The error bars represent the standard errors of the mean (SEM), ns: not significant, and ****: *p* < 0.0001).

It has been known that rheotaxis response occurs within a specific range of flow speeds, as few sperm orient upstream under a weak flow, and most are swept away by a strong flow (El-Sherry et al., 2014; Tung et al., 2014; Zaferani et al., 2018; Ataei et al., 2021). We found that, under a flow rate expected to induce rheotaxis, clustered sperm exhibited a stronger rheotactic response than individually swimming sperm. First, as the flow rate increased to 0.9 and 1.1 *μ*L/min (Figure 3B), it could be seen that clustered sperm were more oriented against the flow than were individual sperm (*p* < 0.0001 by two tailed *t*-test). To further illustrate the effects, three measures were used to make this comparison: probability distribution of sperm orientation, upstream components of sperm orientation, and percentage of sperm oriented upstream. We also compared these three measures against no flow as a control to show that the differences found between clustered and individual swimming sperm were results of the externally applied flow. In the absence of flow, the orientation angle distributions in clustered and individual sperm were nearly flat and similar (*p* > 0.05 by two sample K-S test (Figure 4A). In contrast, at 1.5 *μ*l/min flow, the orientation angle distribution of clustered sperm showed a more pronounced peak in the upstream direction (*θ* = 0°) than orientation of individual sperm (*p* < 0.0001 by two sample K-S test, Figure 4B).

In the absence of flow, clustered and individual sperm showed means and medians of *S*_*x*_ that were close to 0 and only slightly different (*p* = 0.0434 by two tailed *t*-test, Figure 4C). Here, the differences in the statistics of *S*_*x*_ between clustered and individual sperm with no flow arose from the larger variability of data among clustered sperm. In the case of individual sperm statistics, *S*_*x*_ = 0 is obtained from averaging across sperm of all orientations. In the case of clustered sperm, each cluster had several sperm oriented in the same direction, therefore several fold more sperm were required to achieve the same level of accuracy as achieved for individually swimming sperm. When we analyzed all the sperm from the same frame, which led to similar numbers of individual and clustered sperm, the mean for clustered sperm fluctuated more than within the individual sperm (Figure 4A). The means and medians of *S*_*x*_ with a 1.5 *μ*L/min flow rate in clustered and individual sperm showed significant differences between them (*p* < 0.0001 by two tailed *t*-test, Figure 4D). These results showed that clustered sperm were more oriented against the flow than individual sperm. Likewise, under no flow, the percentages of sperm swimming to the left (that is, the upstream direction in the device when a flow is applied) were 47 ± 1% (mean ± SEM) for individual sperm vs. 46 ± 1% for clustered sperm (*p* > 0.05 by two proportion z-test, Figure 4E), which was close to 50% of the ideal value when sperm are uniformly distributed in all directions. The percentages of upstream swimming with a 1.5 *μ*L/min flow was 58 ± 1% for individual sperm and 82.1 ± 0.9% for clustered sperm (*p* < 0.0001 by two proportion z-test, Figure 4F). All of the above results show that clustered sperm responded to an intermediate flow by swimming against the flow better than individually swimming sperm.

### Clustering reduced the numbers of sperm swept downstream by strong flows

**FIGURE 5:**
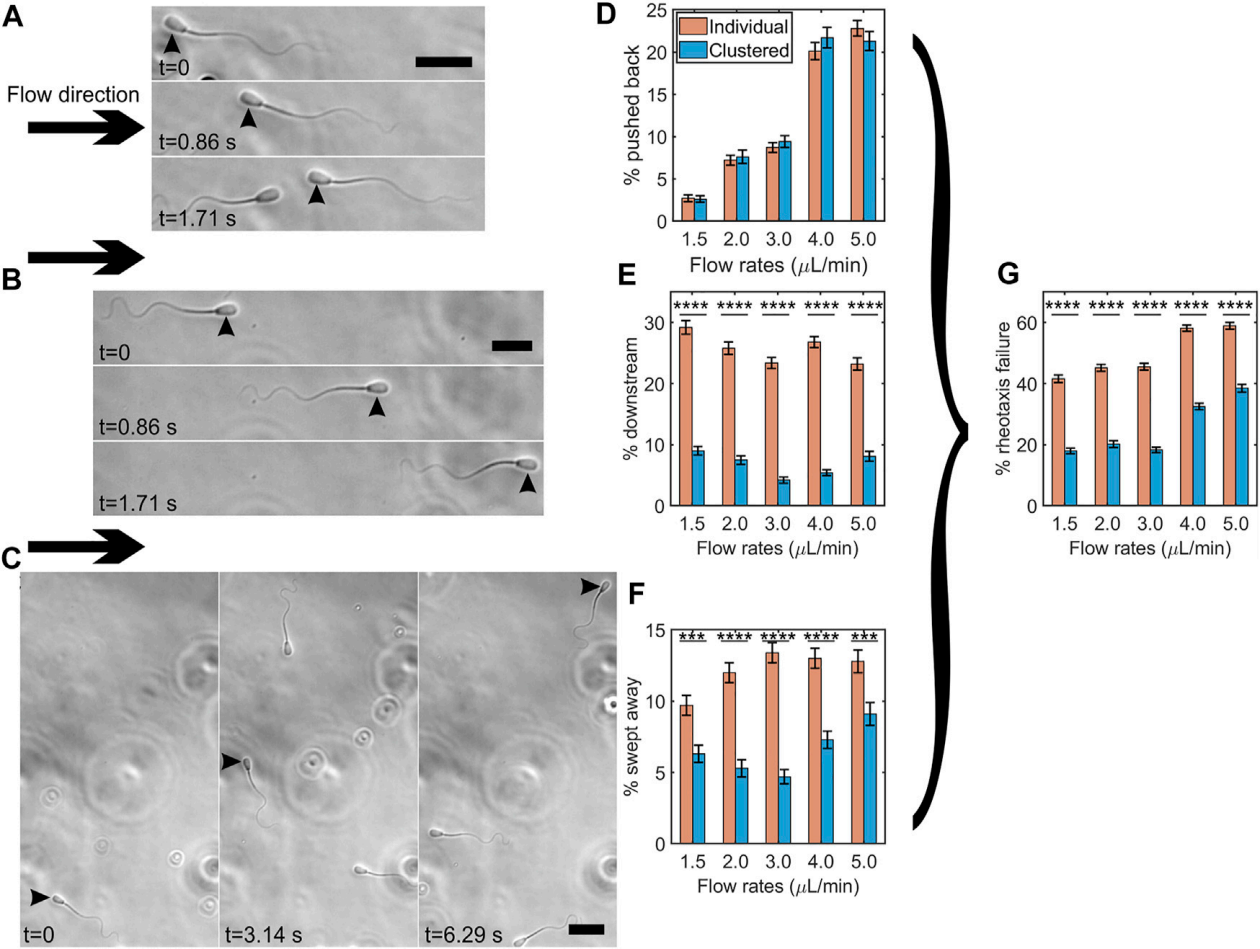
Clustering protected sperm from being swept downstream by a strong flow. Time lapse montage of bovine sperm being pushed back **(A)**, swimming downstream **(B)**, and being swept away **(C)** under a flow of 5.0 *μ*l/min. Flow direction is shown by arrows and the sperm showing the specific behavior is indicated by arrowheads. Scale bar = 25 *μ*m. Percentages of individual vs. clustered sperm being pushed back **(D)**, swimming downstream **(E)**, and being swept away under different flow rates **(F)**. Percentages of individual vs. clustered sperm undergoing rheotaxis failure under different flow rates **(G)**. The error bars represent the standard error of the mean (SEM, N ≈ 3,200–4,300 cells per bar), ***: *p* < 0.001, and ****: *p* < 0.0001. (*p* = 0.91, 0.64, 0.42, 0.21, and 0.30 for 1.5, 2.0, 3.0, 4.0, and 5.0 *μ*l/min respectively for the % of pushed-back sperm by two proportion z-test).

We examined whether clustering protects sperm from being swept downstream by a strong flow. We categorized three types of sperm behaviors when they failed to swim into a strong flow of viscoelastic fluid: (1) sperm that were pushed back by the flow while maintaining upstream orientation (Figure 5A), (2) sperm that swam in the downstream direction (Figure 5B), or (3) sperm that changed orientation from upstream into downstream as they were swept downstream (Figure 5C). Here, we compared the percentages of sperm exhibiting one of the three behaviors that failed in rheotaxis between clustered and individually swimming sperm under a strong flow of 1.5–5.0 *μ*L/min flow rates (or 4.05–13.5 1/sec shear rates) within a time interval of 12 s. Note that we did not observe sperm being swept downstream or pushed back under a flow below 1.5 *μ*l/min.

Figure 5D shows the percentages of pushed back sperm. Unsurprisingly, the percentage increased as the flow rate increased. We did not observe significant differences between clustered and individual sperm. Figure 5E shows the percentages of individual vs. clustered sperm exhibiting downstream swimming. The percentage of individual sperm swimming downstream (range, 23–29%) was significantly greater than that of clustered sperm (range, 4–9%) (*p* < 0.0001 by two proportion z-test), suggesting that clustering promoted sperm upstream orientation under a strong flow. Figure 5F shows the percentages of sperm swept downstream by strong flows. At each flow rate, the percentages of clustered sperm that were swept downstream were significantly lower than those of individual sperm (*p* < 0.001 by two proportion z-test), indicating that clustering protected sperm from being swept downstream.

Combining all three types of failure to undergo rheotaxis (pushed back, downstream swimming, and swept downstream) in Figure 5G, at each flow rate, we found that clustered sperm had roughly a 20% lower rate of failing (*p* < 0.0001 by two proportion z-test). Overall, these results suggested that clustering protects sperm from being moved downstream by fluid flow.

## Discussion

Our results support our hypothesis that clustering of bull sperm increases the progressivity and rheotactic capabilities of sperm swimming in viscoelastic fluids. This indicates that clustering benefits sperm migrating to the egg in the female reproductive tract. Here, we identified the benefits of clustering under three different flow ranges. In the absence of flow, clustering enabled sperm to swim more progressively. Under an intermediate flow that induced upstream swimming, clustering oriented sperm to achieve better rheotactic responses. Under a strong flow that was capable of moving sperm downstream, clustering provided protection for sperm from being carried downstream by the flow. Our results predict that clustered sperm are more likely to swim upstream and are more aligned than individual sperm against flows *in vivo*.

Furthermore, we showed that the current mechanistic model for the emergence of upstream swimming of sperm (Tung et al., 2015a) requires modification in order to account for the behavior of sperm in a flow of a highly viscoelastic fluid. Specifically, in highly viscoelastic fluid, we found a lack of a distinct onset of rheotaxis in sperm. The absence of a distinct onset in upstream swimming could be explained by the variability in sperm circular trajectories in highly viscoelastic fluid. Some sperm in highly viscoelastic fluid swam in linear trajectories, which would cause the onset of upstream swimming to be 0. In low viscosity medium, the origin of the onset of upstream swimming is from circular trajectories of sperm (Tung et al., 2015a). The constant turning that leads to the circular trajectories prevents sperm from aligning against the flow. The flow alignment needs to be strong enough to break this circling in order to lock sperm into a consistently upstream direction. Without the circular trajectory in the first place, any flow alignment is sufficient to orient sperm upstream. Combined with the fact that clustered sperm exhibited more linear (less curved) trajectories than individual sperm, upstream swimming was also triggered more readily for clustered than individual sperm. The above observations can be explained well by the existing mechanistic models (Kantsler et al., 2014; Tung et al., 2015a). However, we also found that, in highly viscoelastic medium, a few sperm swam downstream in linear trajectories. A possible explanation derives from the observations is as follows. Sperm in highly viscoelastic fluid propel themselves via planar flagellar beating (Tung et al., 2017; Walker et al., 2020), while, sperm in low-viscosity fluid commonly rotate along the long axis while swimming. Sperm are also known to swim much closer to a solid surface in a highly viscoelastic fluid than in low viscosity fluid (Nosrati et al., 2015). Altogether, the two-dimensional beating and the closeness of the sperm flagellum to the wall may result in the tail experiencing similar or higher hydrodynamic resistance than that experienced by the head, thereby interrupting the turning of head-to-tail orientation that leads to the curved trajectory. More studies on the hydrodynamic interaction between the sperm head/tail and a solid interface will be needed in order to better understand this phenomenon.

Regarding the linearity (progressivity) of clustered sperm trajectories, it has been known that mouse sperm in the genus *Peromyscus*, which cluster by attaching head-to-head, swim with greater linearity than do individually swimming sperm (Fisher et al., 2014). At the same time, it has been shown that flagellar synchronization can be observed among 2-4 sperm with their heads conjoined together (Woolley et al., 2009), which has been found to enhance swimming velocity over that of individually swimming sperm. Likewise, opossum sperm (*Monodelphis domestica*) physically attach head-to-head to form pairs that swim straighter than individual sperm, and that paired sperm swim efficiently in highly viscous fluids (Moore and Taggart, 1995). Here, we showed that bull sperm swimming collectively without physically attaching to one another are affected similarly as sperm that attach physically to each other. This suggests that enhancement of sperm movement progressivity through collective dynamics may be widely observed across different species, but has not yet been recognized because it is a more subtle behavior than sperm physically joining to one another. The mechanistic understanding of this enhancement remains to be understood, although it is useful to point out that opossum sperm swimming collectively exhibit lower amplitude flagellar bends than individually swimming sperm (Moore and Taggart, 1995), which may lead to less directional change over each beat cycle, therefore a more progressive trajectory. It has also been reported that the directional fluctuation of a cluster of *Peromyscus* mouse sperm is less than that of individual sperm (Fisher et al., 2014), resulting in greater linearity of clustered sperm trajectories.

Results in this study highlight the significance of studying sperm motility in a fluid environment that resembles the mucus sperm naturally encounter in the female reproductive tract (Lai et al., 2009; Tung et al., 2015b). Our results predict that clustered sperm have a better chance to swim upstream and are more aligned than individual sperm against the flow *in vivo*. Although there is strong evidence that rheotaxis provides an effective guiding mechanism for mammalian sperm (Miki and Clapham, 2013; Kantsler et al., 2014; Tung et al., 2015b; Zhang et al., 2016), it has also been known that flows stronger than what sperm can overcome exist *in vivo* (Overstreet and Cooper, 1978). While microgrooves in microfluidic devices that mimic microgrooves in the wall of the cervix have been found to protect bull sperm from being swept downstream by a strong flow (Tung et al., 2014), such microgrooves are not ubiquitous throughout the mammalian female reproductive tract. For example, the endometrium of the mammalian uterus typically lack microgrooves, even though strong flows exist due to muscle contraction (Overstreet and Cooper, 1978; Tung and Suarez, 2021). While comparing the percentages of rheotaxis failure between clustered and individual sperm at different flow rates, we found that individual sperm failed in rheotaxis more often than the clustered sperm, indicating that clustered sperm are better positioned to remain swimming against a strong flow.

The strength of the rheotactic response has been related to male fertility. Human sperm from samples showing greater positive rheotaxis were demonstrated to have more normal morphology and better genomic quality (De Martin et al., 2017; Ataei et al., 2021; Sharma et al., 2022). Recently, it was reported that rheotaxis success of bull sperm was positively correlated with bull fertility (Yaghoobi et al., 2022). Similarly, it has been observed that the selection of rheotactic *Mus musculus* mouse sperm *in vitro* increased fertilization success and quality of early embryonic development (Romero-Aguirregomezcorta et al., 2021). Given our results indicating that clustered sperm have improved rheotactic responses, it would be worth investigating the relationship between clustering of sperm in viscoelastic medium to other types of assessments of male fertility. In addition, whether the directional movement of sperm through collective swimming selects certain genetic traits remains to be seen, although it is interesting to point out that some motility features have been linked to advantages such as DNA integrity (Nosrati et al., 2014; Riordon et al., 2019).

From cervix to oviduct, the fluid that fills the female reproductive tract is viscoelastic in nature (Tung et al., 2015b; Striggow et al., 2020); therefore, sperm encounter the mechanical environment required for forming dynamic clusters *in vivo* (Tung et al., 2017). The other requirement for clustering is a high concentration of sperm, such as concentrations found in semen (Tung et al., 2017; Schoeller et al., 2020). During coitus in humans and cattle, the male deposits semen in the anterior vagina at the entrance to the cervix, where sperm quickly enter viscoelastic cervical mucus flowing out of the cervical canal (Sobrero and MacLeod, 1962). In cattle, it has been documented that the cervical walls are lined with microgrooves that provide preferential pathways for sperm to pass through the cervix into the uterus (Mullins and Saacke, 1989). These pathways also shield sperm from the fastest outflow of mucus, which occurs in the center of the main cervical canal. Because dynamic clustering of sperm enhances rheotaxis of sperm, we propose that clustering assists bull sperm in swimming upstream through the outflow of cervical mucus until they reach the microgrooves (Tung et al., 2015b). Dynamic clustering may also assist sperm upstream swimming in other species and in other regions of the female tract, such as the uterotubal junction that connects the uterus to the oviduct.

Our findings indicate that collective swimming is beneficial for sperm migration, even without the physical attachment of sperm to each other. Compared to individually swimming sperm, we found that clustered sperm show better progressivity during no flow, better rheotactic behavior during an intermediate flow, and more protection against a strong flow. These results elucidate the importance of collective swimming in sperm migration against viscoelastic fluid flow within the female reproductive tract. In addition, this information is useful for designing methods and microfluidics devices for selecting sperm for *in vitro* fertilization.

## Data Availability

The original contributions presented in the study are included in the article/Supplementary Material, further inquiries can be directed to the corresponding author.
