# Supplementary material for: Biological benefits of collective swimming of sperm in a viscoelastic fluid
Source: Front Cell Dev Biol. 2022 Sep 22;10:961623. doi: 10.3389/fcell.2022.961623 (PMC9535079; doi:10.3389/fcell.2022.961623)
Supplement: Supplementary file 3 [file DataSheet1.PDF]

Supplementary Material

1 SUPPLEMENTARY VIDEOS

Descriptions:

Supplementary Video S1: Sperm being pushed back by a 5 μm/min flow. Original video recorded at 6.67 frames per second (FPS) and the video remade at 7 FPS.

Supplementary Video S2: Sperm swimming downstream under a 5 μm/min flow. Original video recorded at 6.67 frames per second (FPS) and the video remade at 7 FPS.

Supplementary Video S3: Sperm being swept away by a 5 μm/min flow. Original video recorded at 6.67 frames per second (FPS) and the video remade at 7 FPS.

2 SUPPLEMENTARY FIGURES

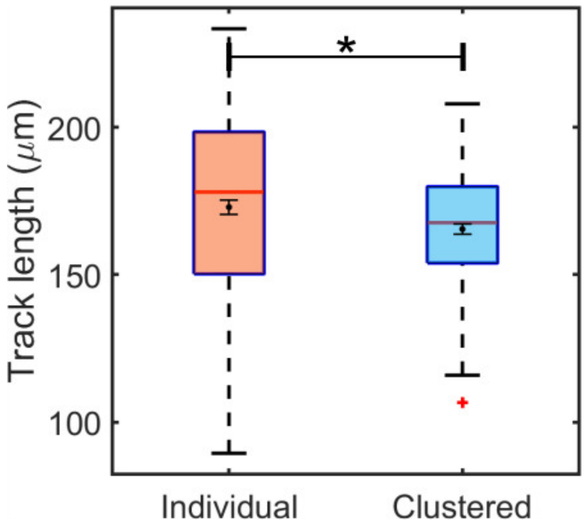

Figure S1. Comparison of track length between individual and clustered sperm (N=150 trajectories). The means are shown by a black dot. The box plot shows the median, 25%, and 75% quartiles; whiskers show the smallest and largest data within 1.5 interquartile ranges below 25% and above 75% quartiles respectively; data beyond the whiskers are outliers and shown by +. Error bars represent standard errors of the mean (SEM). *: $p < 0.05$, t-test.

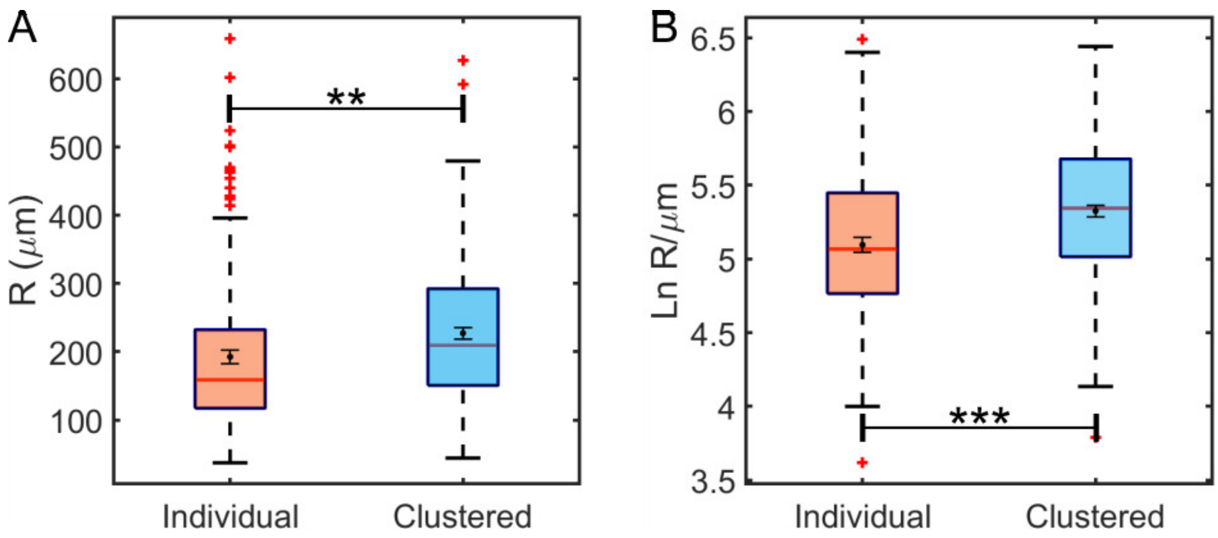

Figure S2. Additional comparisons related to trajectory curvatures between individual and clustered sperm showing the robustness of enhancement in trajectory linearity (N=150 trajectories). (**A**) Radii of curvature (R) and (**B**) the logarithm of the radius of curvature ($\ln R$, geometrical mean)) between individual and clustered sperm respectively. The error bars represent the standard errors of the mean (SEM), **: $p < 0.01$, and ***: $p < 0.001$.

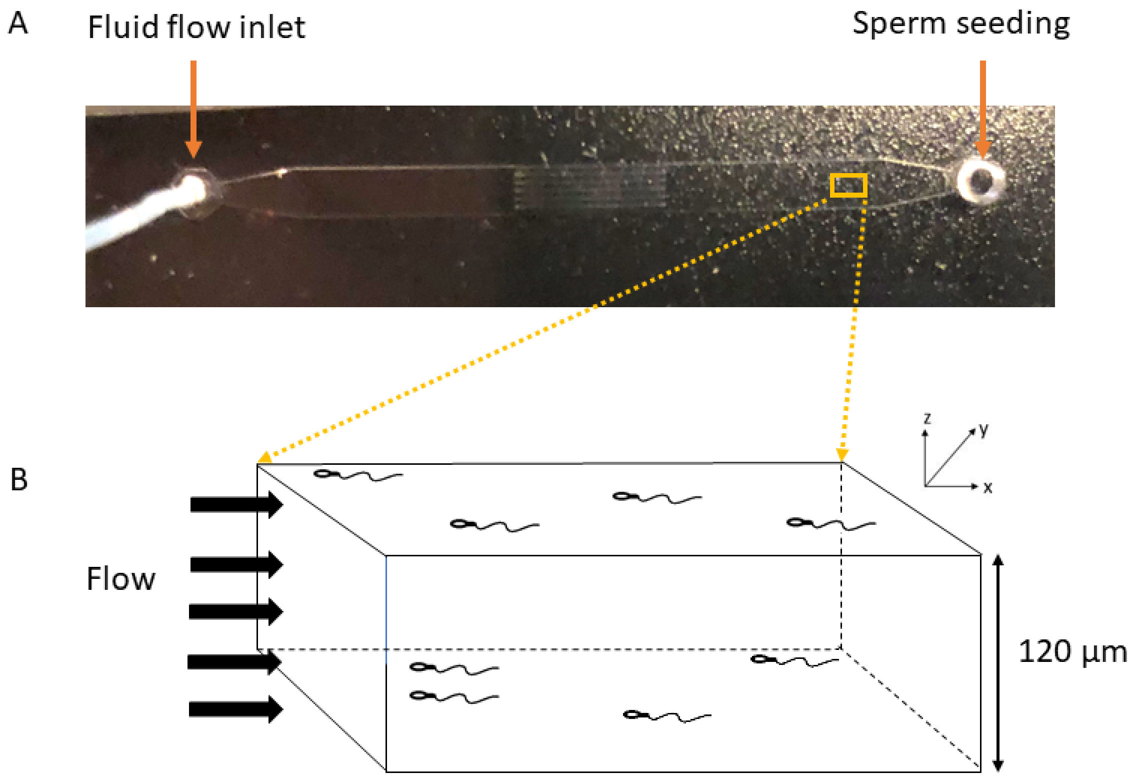

Figure S3. Microfluidic device setup. (**A**) Picture of a PDMS device sealed on glass slide with a sperm seeding port to the right and fluid flow inlet connected to a syringe pump (not shown) to the left through tubing. (**B**) Schematic diagram of sperm swimming in a rectangular microchannel in a field of view indicated in (A) (The sizes are not drawn to scale).
